# Unveiling the Membrane and Cell Wall Action of Antimicrobial Cyclic Lipopeptides: Modulation of the Spectrum of Activity

**DOI:** 10.3390/pharmaceutics13122180

**Published:** 2021-12-17

**Authors:** Roser Segovia, Judith Solé, Ana Maria Marqués, Yolanda Cajal, Francesc Rabanal

**Affiliations:** 1 Section of Organic Chemistry, Department of Inorganic and Organic Chemistry, Faculty of Chemistry, University of Barcelona, 08028 Barcelona, Spain; rosersegovia@ub.edu (R.S.); jsolesol8@alumnes.ub.edu (J.S.); 2 Laboratory of Microbiology, Faculty of Pharmacy and Food Sciences, University of Barcelona, 08007 Barcelona, Spain; ammarques@ub.edu; 3 Department of Pharmacy, Pharmaceutical Technology and Physical Chemistry, Faculty of Pharmacy and Food Sciences, University of Barcelona, 08028 Barcelona, Spain; 4 Institute of Nanoscience and Nanotechnology (IN2UB), 08028 Barcelona, Spain

**Keywords:** antimicrobial peptide, polymyxin, colistin, model membranes, antibacterial resistance

## Abstract

Antibiotic resistance is a major public health challenge, and Gram-negative multidrug-resistant bacteria are particularly dangerous. The threat of running out of active molecules is accelerated by the extensive use of antibiotics in the context of the COVID-19 pandemic, and new antibiotics are urgently needed. Colistin and polymyxin B are natural antibiotics considered as last resort drugs for multi-resistant infections, but their use is limited because of neuro- and nephrotoxicity. We previously reported a series of synthetic analogues inspired in natural polymyxins with a flexible scaffold that allows multiple modifications to improve activity and reduce toxicity. In this work, we focus on modifications in the hydrophobic domains, describing analogues that broaden or narrow the spectrum of activity including both Gram-positive and Gram-negative bacteria, with MICs in the low µM range and low hemolytic activity. Using biophysical methods, we explore the interaction of the new molecules with model membranes that mimic the bacterial inner and outer membranes, finding a selective effect on anionic membranes and a mechanism of action based on the alteration of membrane function. Transmission electron microscopy observation confirms that polymyxin analogues kill microbial cells primarily by damaging membrane integrity. Redistribution of the hydrophobicity within the polymyxin molecule seems a plausible approach for the design and development of safer and more selective antibiotics.

## 1. Introduction

The convergence of antibiotic resistance and the collapse in the antibiotics R&D pipeline calls for urgent action [1,2]. The emergence of multidrug-resistant bacteria is a major global public health concern, and the World Health Organization in its Global Report [3] warns on the consequences of entering a post-antibiotic era. There are different factors associated with the emergence of resistance, and the extensive use of broad-spectrum antibiotics is one of them. In addition, the large pharmaceutical companies have abandoned antibiotic research and development due to economic reasons; the antimicrobial pipeline is getting dry, with the last original class of antibiotic discovered in the late 1980s [1,4,5,6]. The ongoing COVID-19 pandemic has caused an increase in antibiotic use, contributing significantly to the development of resistances [7]. Bacterial co-pathogens and secondary infections are commonly identified in hospitalized, severely ill COVID-19 patients, encompassing between ≈10 and 30% of cases with much greater frequency in the ICU setting [8]. One of the risk factors associated with COVID-19 is secondary bacterial pneumonia, resulting in worst outcomes [9]. In addition, severely ill patients are more likely to receive treatment with invasive catheters, resulting in an increased risk of secondary infections with multidrug-resistant pathogens such as *Acinetobacter baumannii*, *Escherichia coli*, *Pseudomonas aeruginosa*, and *Enterococcus* spp. [10]. These bacteria are among the most threatening pathogens according to the WHO, and effective new drugs are urgently needed. In this regard, several private/public initiatives have been launched in the last years to tackle the problem of antibiotic development. The accelerator program CARB-X (Combating Antibiotic Resistant Bacteria Biopharmaceutical Accelerator) was established in 2016 by partners both from the US and Europe: to July 2021, $396 million have been invested in 92 projects. The Innovative Medicines Initiative launched in 2012 the program New Drugs 4 Bad Bugs (ND4BB) to fuel the development of new antibiotics. So far, €650 million have been invested. Among the various projects within the IMI, ENABLE and AMR ACCELERATOR (IMI2) directly address the development of new antimicrobial compounds. Similarly, the Joint Programming Initiative on Antimicrobial Resistance (JPIAMR) was established in 2011 to consolidate the national research activities within the European Union. [11,12,13].

The lack of new effective antibiotics has forced the recovery of compounds that were left aside in the past. Specifically, polymyxin B and colistin (polymyxin E) have become last-resort antibiotics for the treatment of multidrug-resistant Gram-negative infections, fueling a surge of interest in their use [14,15]. In this context, polymyxins have been extensively studied during the past few years, and great efforts have been made in order to decrease their neuro- and nephrotoxicity and to improve their activity [16,17,18,19].

**Figure 1:**
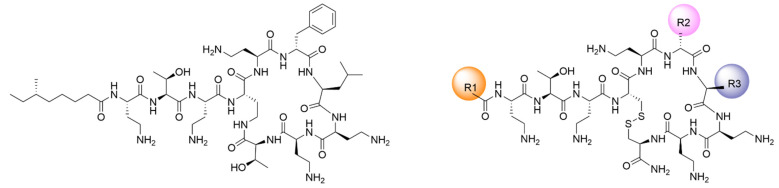
Design of novel polymyxin B analogues. **Left***:* structure of natural polymyxin B; colistin (PxE) has a DLeu in position 6 instead of DPhe. **Right***:* Synthetic scaffold; analogues have a disulfide isosteric insertion to mimic the spatial structure of natural polymyxins. R1: acyl chain C6 to C12; R2: DPhe/DLeu/DNle; R3: Leu/Nle.

Polymyxins are cationic lipopeptides with an amphipatic nature that is critical for their antibacterial activity (Figure 1). The mechanism of action is mainly based on the interaction with the anionic lipids of the bacterial membrane. The hydrophilic domain of polymyxins, with five positively charged amino groups at physiological pH, interacts electrostatically with the negatively-charged lipopolysaccharide (LPS), which is the major component of the outer membrane (OM) of Gram-negative bacteria that is also highly responsible for the packing structure and integrity of these cells. The formation of this complex results in a competitive binding with the membrane-stabilizing divalent cations Mg^2+^ and Ca^2+^. This ion displacement destabilizes the LPS layer, allowing the hydrophobic acyl chain at the N-terminus of the polymyxins to enter through the bacterial membrane. One of the proposed bacterial killing mechanisms is that once in the periplasmatic space, polymyxins form contacts between the outer and inner membranes of the Gram-negative bacteria, which enables a fast exchange of phospholipids that alters the lipid composition of the membrane, thus causing an osmotic imbalance that leads to cell death. It is well known that the cytoplasmic membrane is responsible for many essential functions, including osmoregulation and respiration, and disturbance of the membrane integrity and specific lipid composition can directly or indirectly cause metabolic disfunction and cell death [20,21,22,23,24,25].

The aim of the present work is to explore the role of lipids in the interaction between a series of new analogues of polymyxins and bacterial lipid membrane mimics, with the objective of understanding the molecular basis of the antibacterial mechanism of action. The new compounds, obtained by solid-phase synthesis, have been rationally designed to reduce the toxic effects of natural polymyxins while maintaining antibacterial activity in the context of an ongoing project to develop new antibiotics against resistant bacteria [26,27,28]. Particularly, the contribution of the length of the *N*-acyl chain and of the hydrophobic residues in the cyclic heptapeptide have been evaluated using a synthetic scaffold inspired in natural polymyxins with several key modifications (see the structure in Figure 1).

The interaction of the new lipopeptides with the membrane has been determined using model membranes of different lipid composition to mimic the bacterial cell membranes. Monolayers and liposomes of phosphatidylglycerol (POPG) and mixtures of phosphatidylethanolamine and phosphatidylglycerol, POPE/POPG (6:4) were used as models of Gram-positive and Gram-negative bacteria, respectively; to emulate eukaryotic cells, phosphatidylcholine (POPC) was used. Additionally, in order to simulate the outer membrane of Gram-negative bacteria, monolayers of lipopolysaccharide (LPS) were obtained. Using a combination of biophysical techniques, we have explored the mechanism of action of the lipopeptides in the lipid membrane. Specificity for anionic membranes is a shared characteristic of all the analogues, with no affinity for zwitterionic membranes. Results are consistent with a mechanism of action based on the alteration of the cytoplasmic membrane. In Gram-negative membrane models, all new antimicrobial peptides bind LPS with high affinity and share the ability of natural polymyxins to induce a mixing of anionic lipids between membranes that will result in osmotic imbalance and bacterial death. Some of the analogues are also active on Gram-positive bacteria, and they disrupt the permeability of the cytoplasmic membrane model. Ultrastructural observation of bacterial cells treated with the lipopeptides shows a direct damage on the bacterial envelope that is consistent with the biophysical observations.

## 2. Materials and Methods

### 2.1. Chemicals

1-Palmitoyl-2-oleoyl-*sn*-glycero-3-phospho-(1’-*rac*-glycerol) (POPG), 1-palmitoyl-2-oleoyl-*sn*-glycero-3-phosphoethanolamine (POPE), and 1-palmitoyl-2-oleoyl-glycero-3-phosphocholine (POPC) were purchased from Avanti Polar Lipids (Alabaster, Ala). 1,2-Dipalmitoyl-*sn*-glycero-3-phosphoethanolamine-N-(7-nitro-2-1,3-benzoxadiazol-4-yl) (NBD-PE), 1,2-dioleoyl-*sn*-glycero-3-phosphoethanolamine-*N*-(lissamine rhodamine B sulfonyl) (Rh-PE), 8-aminonaphthalene-1,3,6-trisulfonic acid (ANTS), and *p*-Xylene-bis(*N*-pyridinium bromide) (DPX) were purchased from Invitrogen Molecular Probes (Eugene, OR). 4-Methylbenzydrylamide hydrochloride resin (BHA), N-fluorenylmethoxycarbonyl (Fmoc)-protected amino acids, and trifluoroacetic acid (TFA) were from Fluorochem (Hadfield, United Kingdom) and Iris Biotech GmbH (Marktredwitz, Germany). Hexanoic, heptanoic, octanoic, nonanoic, decanoic, and dodecanoic acids, as well as *N*,*N′*-diisopropylcarbodiimide (DIPCDI), were purchased from Thermo Fisher Scientific (Waltham, MA, USA) and Fluka (Buchs, Switzerland). Polymyxin B and Polymyxin E sulfate salt (PxE and PxB), lipopolysaccharide (LPS) from *Salmonella enterica* serotype Minnesota Re 595 (Re mutant), Trizma base (Tris), and *N*-hydroxybenzotriazole (HOBt) were purchased from Sigma-Aldrich (St. Louis, MO, USA). Chloroform (HPLC grade, Fisher Scientific CO) was used as the spreading solvent for all lipids. All chemicals were of the highest available purity. Water was doubly distilled and deionized (Milli-Q system, Millipore Corp., Burlington, MA, USA).

### 2.2. Peptide Synthesis and Purification

Peptide synthesis was performed manually following standard Fmoc/tBu procedures using 3-fold molar excess of amino acid/DIPCDI/HOBt activation on a Rink amide linker attached via alanine to MBHA resin (f = 0.69 mmol/g of resin). Each coupling was performed in the minimum amount of N, N-dimethylformamide (DMF) for one hour, after which the resin was drained and washed (5 × 1 min) with DMF and methylene chloride. All coupling reactions reached yields ≥99% as assessed by the Kaiser test. The N-terminal Fmoc group removal was carried out by using 20% piperidine in DMF (1 × 1 min, 2 × 10 min). Once the sequence was assembled, cleavage of the peptides from the resin was carried out by acidolysis with TFA/TIS/H_2_O (95:3:2, *v/v*) for 90 min. The cleavage procedure was repeated twice. TFA was removed via N_2_ stream, and the oily residue was treated with dry diethyl ether to obtain the peptide precipitate. The solid peptide was isolated by centrifugation. The homogeneity of peptide crudes was assessed by analytical HPLC on Nucleosil C18 reverse-phase columns (4 mm × 250 mm, 5 µm particle diameter, and 120 Å porous size). Elution was carried out at 1 mL·min^−1^ flow with mixtures of H_2_O/0.045% TFA and acetonitrile/0.036% TFA and UV detection at 220 nm. Cyclization of peptides through disulfide bonds was carried out at high dilution (1 mg/mL) in DMSO/ H_2_O (3:97, *v/v*) for 24–48 h and monitored by analytical HPLC. Peptides were purified by preparative HPLC on a Waters Delta Prep 3000 system using a Phenomenex C18 (2) column (250 mm × 10 mm, 5 µm) eluted with H_2_O/ACN/0.1% TFA gradient mixtures and UV detection at 220 nm. Final purity was greater than 98%. Peptides were characterized by analytical HPLC and ESI mass spectrometry. Peptide stock solutions were prepared in pure water and stored at −25 °C until use. We found no problems related to peptide adherence to Eppendorf^®^ tubes (polypropylene), microtiter plates, or glass vials.

### 2.3. Antibacterial Susceptibility Testing

The minimal peptide concentration required to prevent the growth of a given test organism was determined after incubation overnight at 37 °C and was defined as the MIC. Activity was determined in representative bacteria, *Escherichia coli* ATTC 25922, *Pseudomonas aeruginosa* ATTC 27853, and *Staphylococcus aureus* ATTC 25923, using the broth microdilution method. Serial-two-fold dilutions of peptide (from 0.06 to 32 μg/mL) were performed in Mueller–Hinton broth (MHB) culture medium in sterile 96-well polypropylene microtiter plates inoculated with a final concentration of 5 × 10^5^ CFU·mL^−1^ of bacteria in each well. Bacteria were in the pre-exponential phase. Each determination was done by triplicate. In order to be considered acceptable, the three MIC results have to differ in only in one well, and the result was always given as the higher of the three. Drug-free and microorganism-free wells were used as positive and negative controls, respectively. Bacterial growth was visually determined on the basis of turbidity.

### 2.4. Hemolysis Assays

Hemolytic activity was evaluated on fresh rabbit erythrocytes collected in EDTA collection tubes, as previously described [28]. Briefly, an erythrocite suspension in buffer (10 mM Tris, 80 mM NaCl pH 7.4) was obtained by centrifugation (three times, 10 min at 4000 rpm) and adjusted at an optical density of 1 at 540 nm. Aliquots of 200 µL were incubated for 1 h at 37 °C with the different lipopeptides in 96-well polypropylene microtiter plates and centrifuged (15 min, 5000 rpm). Lipopetide-induced hemolysis was determined by measuring the absorbance at 540 nm of released hemoglobin (*A_sample_*) in the supernatant. Each determination was done by triplicate, and as negative and positive controls, erythrocytes in buffer (*A*_0*%*_) and in distilled water (*A*_100*%*_) were employed, respectively. Hemolysis was calculated according the following equation:

% *hemolysis* = 100 · (*A_sample_* − *A_0%_*/*A_100%_* − *A_0%_*).

### 2.5. Transmission Electron Microscopy (TEM) Observation

To prepare bacterial cells for TEM, an overnight culture (12–16 h) of the same bacterial strains used for MIC determination was grown at 37 °C in TSB. Then, 500 µL of this culture were added to 50 mL of tryptone water for a concentration of ≈10^8^ bacteria·mL^−1^. Aliquots of 10 mL were incubated with the lipopeptide at the desired concentration for 2 h at room temperature, and bacterial cells were collected by centrifugation (12,857 g, 20 min). Samples were fixed with 2.5% glutaraldehyde in phosphate buffer for 2 h at 4 °C, washed with the same buffer, and post fixed with 1% osmium tetroxide in buffer containing 0.8% potassium ferricyanide at 4 °C. Then, the samples were dehydrated for 1 h in acetone, infiltrated in a graded series of Epon resin (Ted pella, Inc., Redding, CA, USA) during 2 days, and finally embedded in fresh Epon resin and polymerized at 60 °C during 48 h. Ultrathin sections were obtained using a Leica Ultracut UCT ultramicrotome (Leica, Vienna, Austria) and mounted on Formvar-coated copper grids. Sections were stained with 2% aqueous uranyl acetate and lead citrate and examined in a JEM-1010 electron microscope (Jeol, Tokyo, Japan).

### 2.6. Large Unilamellar Vesicles Preparation

Large unilamellar vesicles (LUVs) of POPG, POPE/POPG (6:4), or POPC, alone or with the fluorescently labeled phospholipids NBD-PE or Rh-PE, were prepared by the evaporation of a mixture of the lipids and probes in CHCl_3_. The solvent was removed by a gentle stream of nitrogen gas followed by overnight storage under vacuum. The dried films were hydrated with Tris 10 mM pH 7.4 to the desired final lipid concentration and then sonicated in a bath type sonicator (Lab Supplies, Hickesville, NY, Model G112SPIT) until a clear dispersion was obtained (typically 3–5 min). The lipid dispersions were extruded through 2 stacked polycarbonate filters (100 nm pore-size, Millipore, Bedford, MA, USA) using a Liposofast-Basic Extruder (Avestin, Ottawa, ON, Canada) to obtain large unilamellar vesicles. The average diameter was determined by dynamic light scattering with a Malvern II-C Autosizer, with a z-diameter between 111 and 117 nm, and a polydispersity of 0.1. For the ANTS/DPX leakage assay, the dried films were hydrated in 12.5 mM ANTS, 45 mM DPX, and 10 mM Tris pH 7.4. Moreover, they contained 0.2% Rh-PE for monitoring the fluorescence before and after the separation of the unencapsulated material, which was carried out by gel filtration on a Sephadex G-50 column eluted with 10 mM Tris 80 mM NaCl. In this way, the final lipid concentration was calculated taking into account the loss of lipid and the dilution of the sample.

### 2.7. Kinetics of Insertion into Monolayers

Monolayer studies were performed at room temperature (25 °C) on a monolayer system (NIMA Technology, Coventry, UK) by using a cylindrical PTFE (Teflon^®^, Wilmington, DE, USA) trough (5 cm diameter) containing a continuously stirred aqueous phase (12 mL, 10 mM Tris pH 7.4) and a Wilhelmy platinum plate connected to an electrobalance. The system was enclosed in a Plexiglass box to reduce surface contamination, and before each run, the plate and the trough were cleaned with hot water at >70 °C to avoid carryover of lipid or peptide. Monolayers of POPG, POPE/POPG (6:4), POPC, or LPS were formed by applying small drops of the lipid stock solutions in CHCl_3_ on the aqueous subphase with a microsyringe (Hamilton Co., Reno, NV, USA) to achieve a surface pressure of 32 mN·m^−1^. After 10 min, allowing for solvent evaporation, a known aliquot of peptide was added without disturbance of the monolayer via an inlet port, and the surface pressure was continuously monitored.

### 2.8. Light Scattering

The dynamic behavior of liposomes as model membranes was measured as the change in the 90° scattered intensity at 360 nm with 1 nm slit-widths on a SLM-Aminco AB-2 spectrofluorimeter. An aliquot of vesicles of the desired composition was added to the cuvette containing 1.5 mL of 10 mM Tris buffer at pH 7.4 with constant stirring, and then, the peptide stock solution was added successively. The change in scattering was recorded continuously with 2 s resolution. The relative change in the intensity of the scattered light, *δI*, is defined as *(I − I_o_)/I_o_*, where *I_o_* is the scattering without peptide, and *I* is the scattering in the presence of peptide.

### 2.9. Fluorescence Assays for Lipid Mixing

Peptide-induced lipid mixing of vesicles of different composition was investigated in the same spectrofluorimeter, measuring the increase in fluorescence resonance energy transfer (FRET) from an NBD-PE donor population to an Rh-PE acceptor population. Measurements were carried out at 25 °C in 1.5 mL 10 mM Tris buffer pH 7.4 with constant stirring by mixing vesicles containing 0.6% of donor (NBD-PE) with vesicles containing 0.6% of acceptor (Rh-PE), which was followed by titration with peptide from a stock solution. The excitation wavelength was set at 460 nm, and the emission was set at 592 nm with slit widths at 4 nm. The total lipid concentration was 107 μM.

### 2.10. Vesicle Leakage Experiment

To assess the leakage of co-encapsulated ANTS and DPX vesicles due to their interaction with peptides, fluorescence measurements were carried out at 25 °C in 1.5 mL 10 mM Tris buffer pH 7.4 on a SLM-Aminco AB-2 spectrofluorimeter with constant stirring. Lipid vesicles (107 µM) were titrated with lipopeptide from a stock solution 0.25 mM. The excitation wavelength was set at 350 nm, and the emission was set at 530 nm with slit widths at 4 nm. The maximum fluorescence value was obtained by adding 10 µL of triton X-100 to the lipid solution, and it represented the 100% leakage, while the 0% leakage corresponded to the fluorescence of the vesicles at time zero.

## 3. Results and Discussion

### 3.1. New Lipopeptides Inspired in Polymyxins with High Antibiotic Activity

Polymyxins are a class of AMPs with high activity and selectivity for Gram-negative bacteria. Structurally, they consist of a lipodecapeptide macrolactam with a tail-to-side chain amide bond. Important features that are necessary for the antimicrobial activity include the cyclic heptapeptide and the amphipathic nature of the molecule, with five Dab residues that are positively charged at physiological pH, and two hydrophobic domains: the N-terminal lipid tail and the hydrophobic residues DPhe^6^/DLeu^6^ and Leu^7^ in the cycle. In an attempt to maintain the high activity of polymyxins while reducing toxicity, we have designed a chemically accessible scaffold structure that is easily modified to explore individual features, such as the charge, overall hydrophobicity, and amphipathicity, that can modulate the activity/toxicity balance [26]. In this scaffold, as shown in Figure 1, the natural amide bond was substituted by a more chemically accessible link, a disulfide bond between two Cys residues, which is a common structure in many AMPs, such as bactenecin, brevinins, and others. To maintain the natural configuration, we have used an LCys^4^-DCys^10^ link, since this has proven to improve the activity in previous series of analogues [27]. In this study, the antimicrobial activity of PxB and PxE was broadened to include Gram-positive bacteria. We synthesized 16 lipopeptide analogues, nine inspired in PxB and seven in PxE, focusing on the cyclization, charge, and hydrophobicity (Table 1).

Analogues of PxB and PxE in Series I explore the influence of the fatty acid length. Natural polymyxins contain C7–C9 linear or branched fatty acyl tails at the N-terminus [16]. We have substituted them by linear C6 to C12 chains in both PxB and PxE analogues. Series II focuses on the influence of the length of the amino acid side chain in the hydrophobic domain of the cycle, with the substitution of Leu^7^ by Nle. In addition, in the PxE analogues, DLeu^6^ was substituted by DNle or DAoc. Nle has a linear side chain, and we speculated that this may favor the hydrophobic interaction with the phospholipids in the bacterial membrane. The analogue spE-10 contains a 2-D-aminooctanoyl (DAoc) unit in position 6. This amino acid has a lateral side chain that is two methylene units longer than norleucine. The objective is to increase the hydrophobicity in the macrocycle while reducing the one of the fatty acid by shortening it to a heptanoyl (C7) acyl chain. In addition, in this series, an analogue was synthesized incorporating a residue of arginine in place of Dab^8^ (spB-9). Arg is a basic and positively charged amino acid that interacts with high affinity to the anionic phospholipids due to the bidentate cationic character and hydrogen-bond-forming properties of this residue [28].

The results from analogues in Series I show that a minimum length in the hydrophobic N-terminal chain is needed for activity. C6 analogues are devoid of antibacterial effect both against Gram-positive and Gram-negative bacteria, with MICs higher than 32 μg·mL^−1^. C8 analogues show some activity, especially against *P. aeruginosa* but not on Gram-positive *S. aureus*. Good activities are seen in the C10 and C12 analogues, and interestingly, they are active also against *S. aureus*. For example, MIC of 4 μg·mL^−1^ and 2 μg·mL^−1^ against *P. aeruginosa* for spB-3 and spE-3 respectively are obtained, whereas for *S. aureus*, both lipopeptides yield an MIC of 8 μg·mL^−1^. We have previously shown that sp-B, an analogue of PxB with octanoic acid in the N-terminus but with L-Cys^10^, was selective for Gram-negative bacteria [28], and it acted in the same way as PxB on lipid membranes [29]. In this work, analogues are synthesized with D-Cys^10^ to mimic the spatial arrangement of the natural Thr^10^ side chain.

The substitution of Leu by Nle in position 7 (Series II) improves antibacterial activity in all cases, lowering the MIC with respect to the same analogues in Series I. For example, spB-4 and spB-8 have the same C12 acyl chain, and they only differ in the residue in position 7, being Leu or Nle, respectively. MIC is lower in the Nle-containing analogue, with MICs in the order of 1–4 μg·mL^−1^ and maintaining the broad spectrum against both Gram-positive and Gram-negative bacteria. As expected, similar results are obtained with PxE analogues. The mutation Dab/Arg in 8 (spB-9) has little effect on the MIC values. However, spE-10, where the two hydrophobic domains are more balanced, is selective for Gram-negative bacteria, particularly for *P. aeruginosa*, with an MIC of 1 μg·mL^−1^. It is well known that the use of longer fatty acid moieties improves the activity against Gram-positive bacteria [26,30,31], since a long acyl chain allows the insertion of the lipopeptide into the lipid membrane, with the hydrophobic chain parallel to the phospholipid fatty acids allowing a deep insertion and causing membrane destabilization; however, this interaction is less specific and, as it will be discussed later, associated with higher levels of hemolysis and toxicity.

### 3.2. Hemolytic Activity Is Low at the MIC

**Figure 2:**
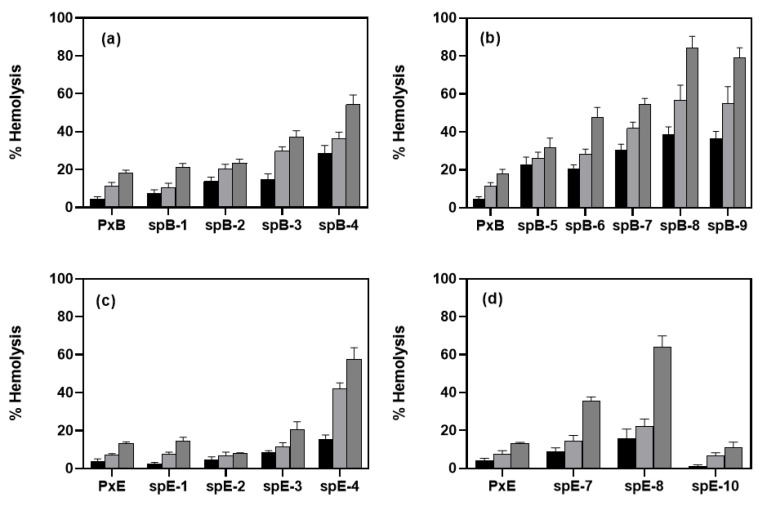
Hemolysis induced by the synthetic analogues of (**a**) Series I-PxB; (**b**) Series II-PxB; (**c**) Series I-PxE; (**d**) Series II-PxE (sequences in Table 1). Erythrocytes were incubated for 1 h at 37 °C with 8 μM (black), 45 μM (light gray), or 335 μM (dark gray) of each lipopeptide. Data correspond to the average of three independent experiments (error bars = SD).

Antimicrobial peptides must be selective for prokaryotic membranes; a well-established method of verifying their ability to damage eukaryotic cell membranes is to measure their hemolytic activity. All of the analogues tested are cationic lipopeptides with an amphipathic nature, which are features that are associated to a non-selective detergent membrane-lytic effect. Hemolytic activity was determined in a wide range of peptide concentrations, from to 8 to 335 μM. In Figure 2, the percentage of hemolysis is shown at three peptide concentrations. All antimicrobial lipopeptides in Series I, both derived from PxB (panel a) or colistin (panel c), are mildly hemolytic at 8 μM, which is a concentration that is representative of the MIC (Table 1), and lysis is comparable to that induced by natural polymyxins. At higher concentrations, hemolysis increases, but even with 335 μM, it is not complete. There is a clear correlation with the fatty acid length, in which C12 analogues are the most hemolytic. This is expected, since the N-terminal acyl chain penetrates the outer membrane, disrupting the highly packed LPS layer, and a long acyl chain is associated with higher toxicity [30,31]. However, C9 and C10 analogues, with good antimicrobial potency, maintain low levels of hemolysis, below 10%. Analogues of PxE (c) are slightly less hemolytic than the corresponding PxB derivatives (panel a), suggesting an effect of DPhe/DLeu^6^ in reducing red cell toxicity. C6 analogues are not hemolytic, but they are also devoid of antibacterial activity. Finally, it seems that the inclusion of a Nle residue increases the hemolytic effect of the peptides derived from PxB and PxE (panels b and d). Interestingly, spE-10 induces very low levels of hemolysis, even below natural colistin. For example, at 8 μM, which is a concentration above the MIC of this molecule against Gram-negative bacteria (Table 1), less than 1% of hemolysis is detected. Even at the highest concentration of 335 μM, only 10% of hemolysis is detected compared to 13% for PxE or 18% for PxB.

### 3.3. Ultrastructural Effects of Lipopeptide Treatment on Bacteria

**Figure 3:**
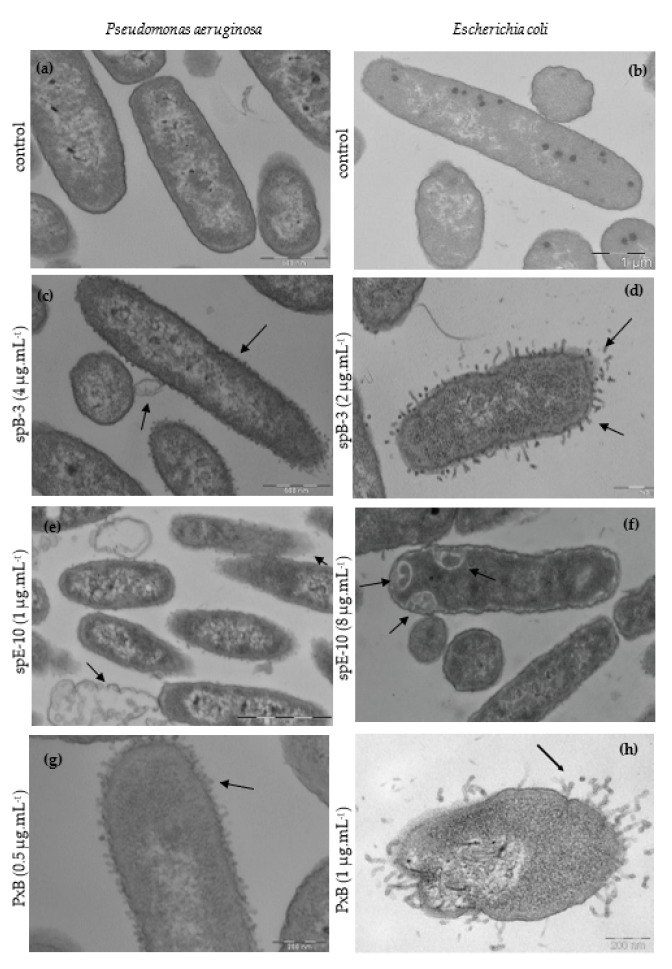
Comparative TEM micrographs of *P. aeruginosa* (left) and *E. coli* (right). Untreated bacteria (**a**,**b**); bacteria treated with polymyxin analogues at 1xMIC: *P. aeruginosa* with spB-3 (4 μg·mL^−1^) (**c**), or with of spE-10 (1 μg·mL^−1^) (**e**); *E. coli* treated with spB-3 (2 μg·mL^−1^) (**d**) or spE-10 (8 μg·mL^−1^) (**f**). Lipopeptides cause the alteration of the membranes, with blebs protruding, loss of membrane, clear cytoplasmic zones, and even ghost cells. Similar results are obtained with natural PxB ((**g**,**h**), this last one is adapted from from [26] (Rabanal et al. *Sci. Rep.* 2015).

Transmission electron microscopy (TEM) of bacteria treated with lipopeptides at the MIC gives information on their impact on the cell membrane. In Figure 3, untreated cells of *P. aeruginosa* (a) and *E. coli* (b) in tryptone water medium showed an undamaged inner membrane, an intact slightly waved outer membrane, and a thin periplasmic space. Incubation of *P. aeruginosa* with 4 µg·mL^−1^ of spB-3, the C10 analogue of PxB, during 2 h, causes the formation of blebs that protrude from the outer membrane (Figure 3c). Blebbing is also observed in *E. coli*; for example, in Figure 3d, cells are treated with spB-3 at 2 μg·mL^−1^, resulting in extensive bleb formation. It is worth noting that spE-10 has a different effect on cell morphology. As shown in Figure 3e, treating *P. aeruginosa* with this analogue even at a concentration of only 1 µg·mL^−1^ (MIC) causes significant rupture of the cell membranes and cytoplasmic clear zones. In *E. coli*, spE-10 induces the formation of intracellular membranous structures and cytoplasmic clear zones (Figure 3f), instead of outer membrane protrusions. Cell envelope blebbing and intracellular damage is also caused by native polymyxins at their MIC (panels g, h in Figure 3) and other AMPs such as gramicidin S [32] or synthetic analogues of PxB [33,34]; for example, the PxB analogue sp-85, including 2 Arg residues, causes severe damage to the cytoplasmic membrane of *E. coli*, with leakage of the cellular components and the formation of membranous inclusions [28]. We have previously shown that analogue spB-7, containing a D-Nle residue and with excellent antibacterial activity, causes important damage on the cell envelope. For example, in *E. coli*, we can see the formation of protrusions or blebs with tubular and fimbria-like radiant appendages on the cell envelope, as described for D-Phe analogues, but in addition, we can see intracellular membranous structures and a swollen periplasmic space (not shown here, but see lipopeptide 38 in [26]).

**Figure 4:**
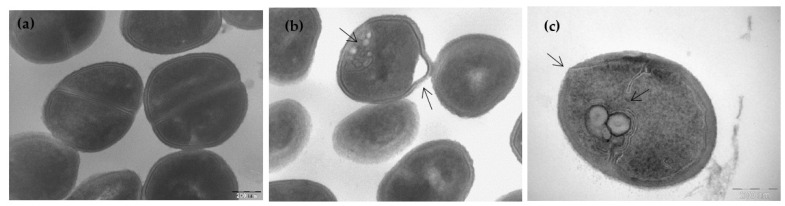
TEM observation of the effect of analogues on *S. aureus*. (**a**) Untreated cells are round and intact, with a well-defined cell membrane. After treatment with 1xMIC of spB-3 (8 μg·mL^−1^) (**b**) or spB-8 (2 μg·mL^−1^) (**c**), clear alterations of the cell morphology are observed, with the formation of mesosome-like structures.

Since some of the polymyxin analogues also have good activities against *S. aureus*, the effects on the cell morphology of this Gram-positive bacteria were determined. In Figure 4, untreated *S. aureus* (a) shows the expected round shape and intact cell envelope. Upon incubation for 120 min with spB-3 at its MIC (8 µg·mL^−1^), we observed the formation of spherical mesosome-like intracellular structures and membranous inclusion bodies together with deformation of the cell envelope (Figure 4b). Bacteria also exhibited cytoplasmatic areas of low electron density attached to the membrane, suggesting a peptide-induced release of cytoplasmic material. Even more disruptive effects were obtained after incubation with the C12/Nle^7^ derivative spB-8 at 2 µg·mL^−1^ (Figure 4c). These effects were previously described for analogue spB-7 (compound 38 in [26]) and are attributed to cytoplasmic membrane alteration caused by the lipopeptides, as they are also induced by other antibiotics [33,34]. For example, AMS NK-2, an internal fragment of mammalian NK-lysin, causes very similar morphological changes in *S. aureus* [35]. The formation of spherical mesosomes is also induced by gramicidin S [32], defensin [36], and cationic peptides *R9F2* and *(KFF)3K* [37], and it is attributed to the lateral membrane expansion caused by insertion of the peptide.

### 3.4. Binding Affinity of the Lipopeptides Is Increased toward Bacterial-Like Model Membranes

**Figure 5:**
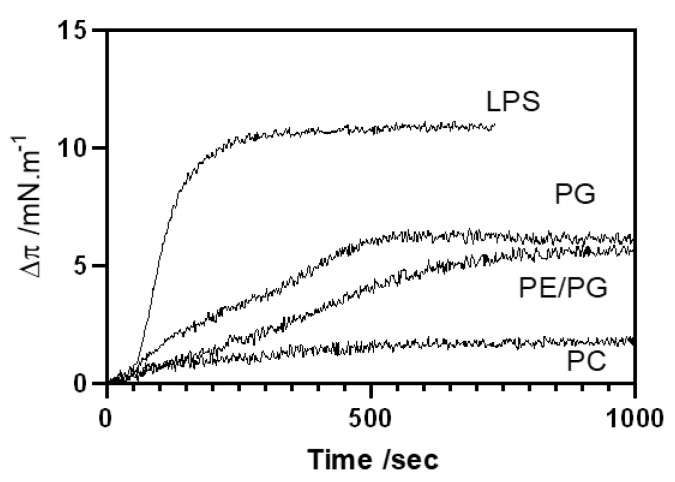
Kinetics of insertion of spB-3 into monolayers of different composition at constant area. Peptide concentration 0.48 μM. Initial surface pressure 32 mN·m^−1^.

In vitro experiments have shown that most of the new synthetic peptides are effective against bacteria and that they have low impact on the eukaryotic red blood cells at their MICs. We next determined the specificity to partition on different lipid membranes using monolayers at the air–water interface. Monitoring insertion of the lipopeptides into lipid monolayers at a constant area is a sensitive tool for studying peptide/lipid interactions, and it provides significant information to understanding the mechanism of action. Monolayers of different compositions (Table 2) were stabilized at 32 mN·m^−1^, which is a surface pressure that is considered equivalent to the natural bacterial membranes [38]. Peptides were injected into the subphase at a concentration of 0.48 μM, which is lower that the MIC, to detect affinity without disruption of the monolayer. In Table 2, the surface pressure increases obtained after 25 min are shown, and a representative experiment with the time-dependent increase in surface pressure is shown in Figure 5.

PxB has very low affinity to bind the eukaryotic plasma membrane, which is mainly due to the lack of electrostatic interaction between the cationic antibiotic and the plasma membrane, which is rich in zwitterionic phospholipids such as phosphatidylcholine (POPC). All of our PxB-analogues share this affinity, with no noticeable or very low pressure change in POPC monolayers (Table 2). This binding selectivity is paramount to reduce toxicity toward mammalian cells [39]. LPS is the component of the outer monolayer of the OM in Gram-negative bacteria. Binding to LPS is a necessary first step for activity, and natural PxB shows high affinity and insertion in the highly packed LPS layer, with an increase in surface pressure of 11.4 mN·m^−1^. All of the synthetic derivatives insert into the LPS monolayer with similar levels of penetration. This is consistent with a mainly electrostatic interaction, since all of the analogues are protonated at pH 7.4 and exhibit the same number of positive charges. Clearly, the contribution of the hydrophobic interaction is necessary for the antimicrobial action but not for LPS binding. For example, we have already shown that PxB nonapeptide, the deacylated derivative of natural PxB, binds to LPS but has no antimicrobial activity [29]. Binding to the cytoplasmic bacterial membrane was assessed with POPG, the main component of Gram-positive membranes, such as *S. aureus*, and a mixture of zwitterionic PE with anionic PG, which is representative of the Gram-negative inner membrane. For example, *P. aeruginosa* has a 6:4 proportion of these phospholipids [38]. No relevant differences were seen with these two lipid compositions, with increases of 4–7 mN·m^−1^ in both monolayer systems, thus indicating that the insertion into the phospholipid layer requires both electrostatic and hydrophobic interactions. In Figure 5, the kinetics of insertion are shown for spB-3, which is the analogue of PxB with a C10 acyl chain. Insertion is very fast for LPS, as also shown with other analogues of PxB [29], but penetration into phospholipid monolayers is more time-dependent, although the maximum increase in pressure is attained in less than 15 min. Similar results were obtained with PxE analogues, except for spE-10. This analogue did insert into LPS with an increase of 14.8 mN·m^−1^ and of 8.2 mN·m^−1^ in PE/PG monolayers, indicating very good affinity and insertion in these two membrane models. Surprisingly, it did not cause any increase in surface pressure when injected into POPG monolayers packed at 32 mN·m^−1^, thus indicating that this lipopeptide did not insert into the anionic monolayer and probably binds only at the lipid surface. Only in the presence of POPE is this analogue able to insert and increase the surface pressure.

### 3.5. Lipopeptide-Induced Aggregation and Lipid Mixing Is Selective for Anionic Vesicles

It is well known that polymyxin B and other AMPs at low concentration in the membrane are able to induce the aggregation of unilamellar vesicles forming clusters. In these clusters, oligomers of the lipopeptide establish discrete contacts and facilitate the selective exchange of phospholipids. This is postulated as the mechanism of action of these AMPs [16,30,40]. Other cationic AMPs cause cell death by direct alteration of the cytoplasmic or inner membrane, forming pores that allow the leakage of intracellular contents or even causing lysis of the bacterial membrane, in what is known as the detergent effect ([41] and references therein). As a first screening to discriminate between these possibilities, we determined the changes in light scattering induced by the peptides on binding to unilamellar vesicles of different composition.

**Figure 6:**
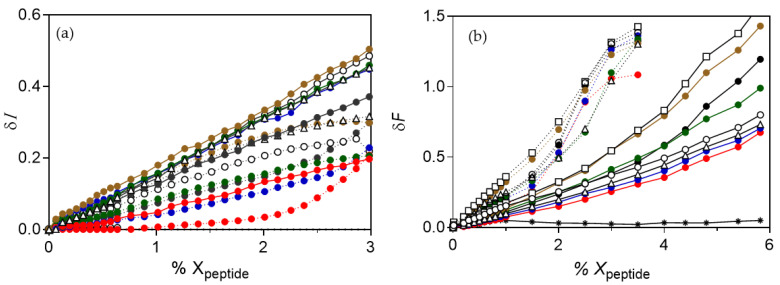
Effect of PxE analogues on unilamellar vesicles of different composition. (**a**) Change in light scattering. (**b**) Increase in FRET intensity as a function of the mole fraction of lipopeptide added to a (1:1) mixture of vesicles containing 0.6% NBD-PE or Rh-PE. Vesicles of POPE/POPG (6:4) (dotted line), POPG (solid line). Peptides are analogues of colistin numbered as in Table 1: spE-1 (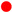), spE-2 (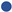), spE-3 (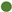), spE-4 (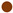), spE-7 (△), spE-8 (◻), spE-10 (◯), or PxE (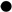). In all cases, no representative changes were detected with POPC vesicles, as shown in (**b**) for PxE (∗). Each data is the mean of three independent experiments, with error being always below 10%.

Upon binding to anionic vesicles, both of POPG and POPE/POPG, all of the analogues induced an increase in scattering, indicating vesicle aggregation, and ruling out the possibility of a detergent or lytic effect. Results for colistin (PxE) analogues are shown in Figure 6a, but similar values were obtained with PxB analogues (not shown). For clarity, only some of the analogues are shown. For a given peptide, the increase is higher for pure PG vesicles than for mixed PE/PG, meaning that bigger aggregates are formed in the Gram-positive membrane model. Aggregation is related to the length of the acyl chain, with higher increases in scattering obtained with C12 analogues and very low aggregating capacity with the C6 analogue with the same amino acid structure; however, in no case did we see a decrease in particle size. None of the analogues had any effect on the zwitterionic POPC vesicles, with no changes in light scattering even at 3 mol % of each peptide.

We next determined the possibility of lipid mixing between the aggregated vesicles by monitoring the increase in FRET when vesicles containing 0.6% NBD-PE (donor vesicles) were mixed with vesicles containing 0.6% Rh-PE (acceptor vesicles) (Figure 6b). All lipopeptides induced the mixing of lipids between anionic vesicles of PG and PE/PG but not between the zwitterionic PC vesicles, which is consistent with the scattering results. Surprisingly, the increase in FRET signal for a given analogue was higher on the PE/PG vesicles compared to the PG ones. These results, together with the scattering increase, indicate that even though smaller clusters are formed in the mixed PE/PG vesicles, the number of clusters formed is high. A similar behavior was previously reported for synthetic Arg-containing analogues of PxB; it also induced lipid mixing selectively in anionic vesicles, which is an effect that was potentiated by the presence of the zwitterionic lipid PE found in Gram-negative membranes [27].

### 3.6. Leakage of Aqueous Contents Depends on Lipid Composition

**Figure 7:**
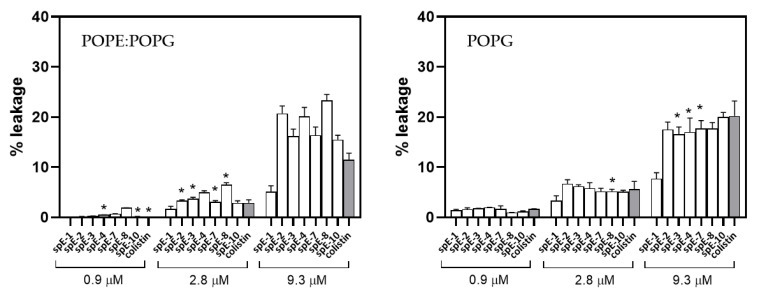
Leakage from vesicles of POPE/POPG (**left**) or POPG (**right**) at three peptide concentrations. Lipopeptides were added to liposomes co-encapsulating ANTS (12.5 mM) and DPX (45 mM), and leakage was determined as the increase in ANTS fluorescence intensity at 530 nm (excitation 350 nm, lipid concentration 107 µM). Data represent the mean ± SD (n = 3). Concentrations close to the MIC are marked with an asterisk.

Once demonstrated that the antibacterial lipopeptides induced the aggregation and lipid mixing between anionic vesicles, the next question is if this is also accompanied by the leakage of aqueous contents, indicating membrane fusion events. This was assessed by measuring the increase in fluorescence of ANTS co-encapsulated with the fluorescence quencher DPX, which was induced by the addition of each lipopeptide from a stock solution. In Figure 7, leakage at three relevant concentrations of the analogues of colistin is shown, and the concentration that more closely resembles the MIC is marked with an asterisk. In membranes of PE/PG (6:4), modeling the IM of Gram-negative bacteria, leakage is low in all cases, and at the concentrations corresponding to the MIC of each antibiotic remains below 5%, even though at the same lipid-to-peptide ratio, there is significant aggregation and lipid mixing. Even at the high peptide concentration of 9.3 µM, which corresponds to 8 mol % peptide, leakage is lower than 25%. Colistin behavior is similar, with low induction of leaky fusion even at high concentrations, as was also previously demonstrated for PxB [27]. The low permeabilization effect is also seen with the most active peptides with low MICs for Gram-positive and Gram-negative bacteria: for example, spE-4 analogue, with a C12 acyl chain able to insert deeply into the bilayer, and with MICs of 1 µg·mL^−1^ for P. aeruginosa and 8 µg·mL^−1^ for *S. aureus*, or spE-7, a very active broad spectrum molecule that also causes very low membrane permeabilization. Special attention deserves spE-10, since this analogue maintains the specificity for Gram-negative bacteria that is characteristic of polymyxins. spE-10 causes no detectable leakage from PE/PG vesicles at 0.9 µM (corresponding to 0.8% peptide mol fraction), which is slightly higher than the MIC of this analogue on P. aeruginosa. These results are consistent with the formation of vesicle–vesicle contacts, forming clusters of vesicles (aggregation), and the mixing of lipids from the outer monolayers of the vesicles in contact, but without causing permeabilization of the inner compartments. This is the mechanism of action of natural polymyxins and other antimicrobial peptides, inducing cell death by the loss of compositional specificity in the IM and OM of Gram-negative bacteria, causing osmotic shock.

In the case of POPG vesicles, the permeabilization effect is also concentration-dependent and negligible at low concentrations. At the highest peptide concentration of 9.3 µM, it remains below 20% and is comparable to colistin. Analogue spE-1, with a C6 acyl chain and no antibiotic effect, has the lowest permeabilizing effect, even though it can induce aggregation and lipid mixing (Figure 6).

Finally, leakage from POPC vesicles is negligible in all cases (not shown).

## 4. Conclusions

To improve our knowledge of the mechanism of action of antimicrobial peptides, it is necessary to design new molecules with better therapeutic properties, combining excellent activity with minimal toxic effects on the host. Bacterial membrane biophysics can contribute, providing relevant information on the interaction with the membrane at the molecular level [42,43], since model membranes are well recognized systems with demonstrated biological relevance [44]. Combining biophysical studies with in vitro activity on bacteria, observation of alterations in bacterial membrane integrity by electron microscopy, and induction of hemolysis, we provide a way to screen new molecules for further studies. With this approach, we report new synthetic potent antimicrobial peptides inspired in natural PxE and PxB, which are two last resort antibiotics that are very potent and selective for Gram-negative bacteria but toxic. We have shown that it is possible to fine-tune the antibacterial spectrum of activity by modification of the hydrophobicity within the molecule. For example, some compounds showed a broad spectrum of action including Gram-positive and Gram-negative bacteria, together with low levels of induction of hemolysis. We demonstrate that increasing the length of the N-terminal acyl chain above C8 results in relevant antibacterial activity not only on *P. aeruginosa* and *E. coli* but also against *S. aureus*. Combining this longer acyl chain with a point mutation to increase the length of the side chain of the hydrophobic residue Leu^7^ results in new, very potent, broad-spectrum antimicrobial lipopeptides. For example, spE-7, with a C10 acyl chain and two Nle residues has MICs of 2 µg·mL^−1^ (1.6 µM) and 8 µg·mL^−1^ (6.7 µM) for *P. aeruginosa* and *S. aureus*, respectively, and it causes less than 10% hemolysis at 8 µM. We also described that a shortening of the acyl chain length to C7, together with increasing the apolar character of the hydrophobic domain of the peptide cycle, results in a molecule, spE-10, that is selective for Gram-negative bacteria, particularly *P. aeruginosa*, and also has the lowest level of hemolysis, including natural colistin (PxE). Interaction of the lipopeptides with model membranes indicates selective binding to anionic LPS, a model of Gram-negative OM, or to specific phospholipids that mimic the IM of Gram-negative and Gram-positive bacteria, with the formation of clusters of vesicles and induction of lipid exchange starting at very low peptide concentrations. Low levels of leakage induced by the peptides at the relevant concentrations needed for activity suggest that the mechanism of action is not based on bilayer permeation by channel formation. Lipopeptide-induced bacterial membrane alteration is confirmed by electron microscopy observation, with the formation of blebs and other membrane alterations on Gram-negative bacteria, in a similar way to that of natural polymyxins, or the formation of intracellular mesosomes in Gram-positive *S. aureus*. The interaction of spE-10 with model membranes is consistent with its selectivity toward Gram-negative bacteria. This analogue requires the presence of PE in order to insert into monolayers packed at the membrane-equivalent pressure. PE is a phospholipid abundant in the cytoplasmic membrane of Gram-negative bacteria, it is zwitterionic and has a cone-shaped form, and it plays an important role in the interaction of cationic AMPs with the membrane [27]. The interaction of spE-10 with anionic liposomes shows aggregation and lipid mixing, but no significant leakage at the relevant concentrations needed for antimicrobial activity, and TEM images of treated Gram-negative bacteria also show important membrane damage.

In summary, we have shown that the modulation and redistribution of the hydrophobicity within our polymyxin disulfide scaffold allows fine-tuning antibacterial activity. In particular, such reorganization in analogue spE-10 confers selectivity toward *P. aeruginosa*. This is an encouraging result, since selectivity in antibacterial molecules is a potentially advantageous characteristic in the development of antibiotics, particularly against *P. aeruginosa*. Infections caused by this type of bacteria are considered a serious health threat both by the CDC and the ECDC [45,46]. In this sense, murepavadin (POL7080), a cyclic peptide that specifically kills *P. aeruginosa*, reached clinical phase III and it is now being evaluated as an inhalation therapy for the treatment of cystic fibrosis [47]. Altogether, the low MIC value shown by spE-10 against *P. aeruginosa* combined with its lower hemolytic activity compared to colistin makes this analogue an attractive hit compound to start development. In summary, redistribution of the hydrophobicity within the polymyxin scaffold seems a plausible approach for the design and development of safer and selective antibiotics.

## Figures and Tables

**Table 1:** Sequences and antimicrobial activities of the synthesized peptides determined as minimal inhibitory concentration (MIC) calculated for different bacteria strains.

		Sequence	MIC/µg mL^−1^ (µM)		
			EC ^1^	PA	SA
Series I-PxB	spB-1	hexanoyl-Dab-Thr-Dab-Cys-Dab-*Phe*-Leu-Dab-Dab-*Cys*	>32 (>27)	>32 (>27)	>32 (>27)
	spB-2	octanoyl-Dab-Thr-Dab-Cys-Dab-*Phe*-Leu-Dab-Dab-*Cys*	16 (13)	8 (6.6)	>32 (26)
	spB-3	decanoyl-Dab-Thr-Dab-Cys-Dab-*Phe*-Leu-Dab-Dab-*Cys*	2 (1.6)	4 (3.2)	8 (6.5)
	spB-4	dodecanoyl-Dab-Thr-Dab-Cys-Dab-*Phe*-Leu-Dab-Dab-*Cys*	16 (13)	8 (6.3)	8 (6.3)
Series II-PxB	spB-5	octanoyl-Dab-Thr-Dab-Cys-Dab-*Phe*-Nle-Dab-Dab-*Cys*	2 (1.7)	4 (3.3)	>32 (>26)
	spB-6	nonanoyl-Dab-Thr-Dab-Cys-Dab-*Phe*-Nle-Dab-Dab-*Cys*	2 (1.6)	1 (0.82)	16 (13)
	spB-7	decanoyl-Dab-Thr-Dab-Cys-Dab-*Phe*-Nle-Dab-Dab-*Cys*	2 (1.6)	1 (0.81)	4 (3.2)
	spB-8	dodecanoyl-Dab-Thr-Dab-Cys-Dab-*Phe*-Nle-Dab-Dab-*Cys*	4 (3.2)	1 (0.79)	2 (1.58)
	spB-9	dodecanoyl-Dab-Thr-Dab-Cys-Dab-*Phe*-Nle-Arg-Dab-*Cys*	4 (3.0)	4 (3.0)	2 (1.5)
Series I-PxE	spE-1	hexanoyl-Dab-Thr-Dab-Cys-Dab-*Leu*-Leu-Dab-Dab-*Cys*	>32 (>28)	>32 (>28)	>32 (>28)
	spE-2	octanoyl-Dab-Thr-Dab-Cys-Dab-*Leu*-Leu-Dab-Dab-*Cys*	16 (14)	4 (3.4)	>32 (>27)
	spE-3	decanoyl-Dab-Thr-Dab-Cys-Dab-*Leu*-Leu-Dab-Dab-*Cys*	8 (6.7)	2 (1.7)	8 (6.7)
	spE-4	dodecanoyl-Dab-Thr-Dab-Cys-Dab-*Leu*-Leu-Dab-Dab-Cys	8 (6.5)	1 (0.81)	8 (6.5)
Series II-PxE	spE-7	decanoyl-Dab-Thr-Dab-Cys-Dab-*Nle*-Nle-Dab-Dab-*Cys*	2 (1.7)	2 (1.7)	8 (6.7)
	spE-8	dodecanoyl-Dab-Thr-Dab-Cys-Dab-*Nle*-Nle-Dab-Dab-*Cys*	4 (3.3)	4 (3.3)	4 (3.3)
	spE-10	heptanoyl-Dab-Thr-Dab-Cys-Dab-*Aoc*-Nle-Dab-Dab-*Cys*	8 (6.7)	1 (0.84)	>32 (>27)
Controls	PxB	R1-Dab-Thr-Dab-Dab-Dab-*Phe*-Leu-Dab-Dab-Thr	1 (0.83)	0.5 (0.42)	>32 (>27)
	PxE	R1-Dab-Thr-Dab-Dab-Dab-*Leu*-Leu-Dab-Dab-Thr	1 (0.86)	0.5 (0.43)	>32 (>27)

^1^ EC: *Escherichia coli*; PA: *Pseudomonas aeruginosa*; SA: *Staphylococcus aureus*. Peptide sequences are displayed in three-letter code, D-amino acids are denoted in italics, and underlined residues denote bond formation. R1: (S)-6-methyloctanoyl; MIC for lipopeptides spB-3, spB-7, spB-8 are taken from [26] and correspond to analogues #37, #38, and #39.

**Table 2:** Increase in surface pressure (Δπ/mN·m^−1^) upon penetration of the antimicrobial lipopeptides into monolayers ** at an initial pressure of 32 mN·m^−1^.

	Series I-PxB				Series II-PxB					Series I-PxE				Series II-PxE			
Composition *	spB-1	spB-2	spB-3	spB-4	spB-5	spB-6	spB-7	spB-8	spB-9	spE-1	spE-2	spE-3	spE-4	spE-7	spE-8	spE-10	PxB /PxE
LPS	12.1	12.6	11.0	12.2	10.6	10.8	9.3	11.2	11.1	10.6	9.5	10.0	11.4	14.2	12.5	14.8	11.4/11.1
POPE/POPG	4.6	5.5	6.0	5.0	6.5	5.0	6.5	6.2	6.0	4.1	6.4	7.9	9.1	6.8	9.8	8.2	7.2/7.9
POPG	5.7	7.0	6.2	6.7	7.3	6.0	4.2	3.8	3.4	2.8	4.3	5.7	5.8	8.2	7.4	0.8	5.9/5.3
POPC	0.9	1.8	2.0	2.5	0.8	0.4	2.1	1.7	2.2	0.3	0.6	1.3	2.1	1.4	1.3	1.8	1.4/0.8

* Monolayers mimic the outer membrane of Gram- (LPS), the cytoplasmic membrane of Gram- (POPE/POPG 6:4), or of Gram+ (POPG), or the eukaryotic membrane (POPC). ** Increase in pressure measured 25 min after peptide injection. Peptide structures in Table 1. Results are the average of three independent experiments (error < 10%).

## Data Availability

Not applicable.
